# Factors Involved in Host Resilience to Enteric Infections in Pigs: Current Knowledge in Genetic, Immune, and Microbiota Determinants of Infection Resistance

**DOI:** 10.3390/genes17010067

**Published:** 2026-01-06

**Authors:** Alejandro Ucero-Carretón, Héctor Puente, Marie Ithurbide, Jordi Estellé, Ana Carvajal, Héctor Argüello

**Affiliations:** 1 Departamento de Sanidad Animal, Facultad de Veterinaria, Universidad de León, 24071 León, Spain; aucec@unileon.es (A.U.-C.); amcaru@unileon.es (A.C.); hector.arguello@unileon.es (H.A.); 2 Instituto de Desarrollo Ganadero y Sanidad Animal (INDEGSAL), Universidad de León, 24071 León, Spain; 3 Departamento de Anatomía y Anatomía Patológica Comparadas y Toxicología, UIC Zoonosis y Enfermedades Emergentes ENZOEM, Universidad de Córdoba, 14071 Córdoba, Spain; 4 GABI, Institut National de Recherche pour l’Agriculture, l’Alimentation et l’Environnement (INRAE), AgroParisTech, Université Paris-Saclay, 78350 Jouy-en-Josas, France; marie.ithurbide@inrae.fr (M.I.); jordi.estelle@inrae.fr (J.E.)

**Keywords:** diarrhoea, diseases, intestine, pathogen, genetic editing, virus, bacteria, swine

## Abstract

Enteric infections remain a major health and economic challenge in swine production, with outcomes determined not only by pathogen virulence but also by the complex interplay between host genetics, immune competence, and the intestinal microbiota. This review synthesises current knowledge on host–pathogen genomic interactions in pigs, with a focus on resilience mechanisms against enteric diseases in swine. For this purpose, 103 articles were used as information sources, retrieved through structured keyword searches in PubMed. The review first addresses host genetic factors, highlighting genomic variants and quantitative trait loci associated with resistance or resilience to viral and bacterial pathogens such as porcine epidemic diarrhoea virus (PEDV) or *Escherichia coli*. Next, the key factors of the immune system to confer protection are also reviewed, emphasising the role of innate and adaptive responses in controlling each pathogen and disclosing the contribution of regulatory networks that balance pathogen clearance. Finally, the last section of the review is devoted to exploring current knowledge in the involvement of the microbiota in resilience against enteric pathogens, mostly, but not exclusively, enteric bacteria. In this sense, competitive exclusion is a concept which has gained attention in recent years. The review pinpoints and discusses the state of the art about how the microbial community provides colonisation resistance, shapes immune development, and influences pathogen fitness within the intestinal niche. As final perspectives, the review explores future drivers in the genetic immune and microbiota resistance. By bridging host genomic data with functional insights into immunity and microbial ecology, this review underscores the potential of multi-omics approaches to enhance resilience against enteric infections in pigs and advance sustainable swine health management.

## 1. Introduction

Enteric diseases persist as one of the principal health challenges impacting swine production globally. They lead to substantial economic losses through increased mortality, reduced growth performance, and the subsequent associated control costs [1]. Aspects such as the emergence of novel pathogens such as *Brachyspira hampsonii* [2,3] or the porcine deltacoronavirus (PDCoV) [4] are now disturbing gut health in pig farms in America and Asia. Not only have new pathogens appeared, but old suspects such as *Brachyspira hyodysenteriae* [5] or the porcine epidemic diarrhoea virus (PEDV) [6] have also re-emerged and are currently distributed worldwide. Indeed, PEDV outbreaks in America in the early 2010s are an example of the timeliness of enteric diseases in the pork sector [7]. Furthermore, there is an increasing demand of *Salmonella* control in pork products to guarantee pork food safety worldwide [8,9]. Recent policies to reduce and limit antimicrobials worldwide, either as growth promoters in countries such as Brazil or USA [10,11], or current policies in Europe for antimicrobial use reduction and the ban of therapeutic zinc oxide [12,13,14], increase the frequency and the impact of enteric infections which were under relative control before.

Novel and disruptive strategies are needed to improve intestinal health and mitigate the impact of enteric infections. Although alternatives to antibiotics such as novel feed additives, vaccines, or management strategies have been tested, host resistance to infections remains promising but yet under-explored [15,16]. Recent evidence in the development of pigs resilient against the porcine reproductive and respiratory virus (PRRSv) [17] or the classical swine fever virus [18] demonstrates the potential of the host genome to identify and select genetic resilient traits. Host resistance is not only associated with pathogen receptors but also with the capacity to elicit an early and efficient immune response [19]. Last but not least, the microbiota, which is part of the holobiont, plays a crucial role in susceptibility to enteric infections [20]. Moreover, understanding enteric disease resilience requires an integrated epidemiogenetic approach that bridges individual host genetics with population-level disease dynamics. Host genetic variation not only determines individual susceptibility but also influences transmission rates, pathogen evolution, and the effectiveness of control strategies at the population level [21]. This epidemiogenetic framework is essential for developing sustainable disease control strategies that account for both host genetic diversity and pathogen evolutionary responses. This review summarises the current state of the art on these three pillars for pig resilience against enteric pathogens. The article is divided into four sections referring to genetic resistance to bacterial and viral diseases, current knowledge in key aspects of the immune response against the main enteric pathogens, the evidence of the competitive exclusion exerted by the microbiota against enteric pathogens, and finally prospects for research in this field.

## 2. Materials and Methods

### 2.1. Protocol Development

A review protocol was developed using an AI-assisted workflow (ChatGPT, OpenAI, version 5.1). The system was provided with the main research domains (genetic, immune, and microbiome-mediated resistance to enteric pathogens in swine) and generated specific topic sets of controlled vocabulary and free-text terms that were subsequently used to build the search strategy. The strings obtained were manually revised and curated before they were used.

### 2.2. Search Strategy

The search combined controlled vocabulary and free-text terms related to swine, host resilience, and enteric disease, integrating three concept blocks: genetics, immune mechanisms, and microbiome-mediated resistance. The complete search strings used, with all codes intact, are provided in Figure S1. General descriptors included combinations of “swine”, “pigs”, and “porcine”, together with resilience-related terms such as “resilien*”, “toleran*”, “robust*”, and “resist*”, and enteric disease indicators including “enteric”, “intestinal”, “gut”, “gastrointestinal”, and “diarrh*”.

For the genetics domain, additional terms captured genomic approaches and genetic variation, including “genetic*”, “genomic*”, “heritab*”, “GWAS”, “QTL”, “SNP*”, and “polymorph*”. The immune domain incorporated terms representing host defence processes, such as “immune*”, “mucosal immun*”, “innate immun*”, “adaptive immun*”, “IgA”, “cytokine*”, and “disease tolerance”. The microbiome domain included descriptors related to gut microbial communities and modulation strategies, such as “microbiota”, “microbiome”, “probiot*”, “prebiot*”, “synbiot*”, “fecal microbiota transplant*”, and “FMT”. All three domains were systematically combined with pathogen-specific terms, including “*E. coli*”, “ETEC”, “*Salmonella*”, “*Lawsonia intracellularis*”, “*Brachyspira*”, “*Clostridium perfringens*”, “Rotavirus”, “PEDV”, “TGEV”, “PDCoV”, “SADS-CoV”, “coccid*”, “*Cystoisospora suis*”, and “*Eimeria*”.

No language restrictions were applied. In addition to the database search, the bibliographies of included articles and relevant reviews were manually screened to identify further eligible studies. Searches were conducted in PubMed for the period 2000–2025.

### 2.3. Eligibility Criteria

We included studies of any design that investigated host resilience to enteric pathogens in swine through genetic factors, immune mechanisms, or microbiome-mediated enteric pathogen resistance with no restrictions in methodology or publication format.

### 2.4. Study Selection

All retrieved records underwent title and abstract screening followed by the full-text assessment. The searches yielded 209 records for the genetics, 246 for the immunity, and 214 for the microbiome. After applying the eligibility criteria, a total of 39, 39, and 25 studies for each topic/domain, respectively, were finally used as the information source.

## 3. Genetic Background as Basis of Infection Resistance in Enteric Pathogens

The intestine is a component of the digestive system, and its primary function is the digestion and absorption of nutrients. It is lined by a single layer of epithelial cells, composed predominantly of enterocytes, along with other specialised cell types such as M cells and goblet cells. The structure of this surface layer varies from the duodenum to the rectum and is also impacted by the host genetic background, which influences cell receptors, immunity pathways, and gut structure, modifying the susceptibility to different pathogens.

### 3.1. Enterotoxigenic E. coli

Research in the mechanisms of genetic resistance to enteric swine pathogens have been extensively studied and particularly detailed for enterotoxigenic *E. coli* (ETEC). This pathotype of *E. coli* is considered the main pathogen in neonatal and postweaning diarrhoea (PWD) infections [22]. Laboratory classification of ETEC is currently based on its major virulence factors, the fimbriae and enterotoxins, which define virotypes based on the factors present [23].

It is well known that the virotypes affecting pigs in neonatal and PWD infections differ as a result of changes in the expression of the fimbriae receptors by the enterocytes. It has long been established that the adherence to the epithelium by ETEC is only possible when the receptor is expressed on the surface of the enterocyte brush border. Interestingly, while during farrowing, pigs are susceptible to F4 (K88), F5 (K99), F6, and F41 fimbriae, only receptors for F4 and F18 fimbriae are expressed after weaning [24]. A more detailed review of fimbriae receptor expression can be found elsewhere [22,25]. This physiological switch in the expression of receptors may be considered the first level where the host genetic background can influence the gut barrier against ETEC infections, but the mechanism of infection resistance goes beyond it. The main genes involved in ETEC resistance are summarised in Table 1.

It is worth mentioning the thorough research on the genetic basis of resistance against F4 ETEC fimbriae. This factor of adherence consists of two subunits, with three serological types: F4ab, F4ac, and F4ad [26]. Each F4 subtype exhibits a specific binding pattern leading to a complex interaction with the host [27,28]. Fine mapping of the genetic regions linked to the F4ab/ac receptor pointed out the region between *S0068* and *Sw1030* microsatellites in pig chromosome 13. Several genes within this chromosomic region have been proposed as candidates for resistance including mucins and transferrin-like proteins [29,30,31]. More recently, the *HEG1* and *ITGB5* genes have been identified as candidates for F4ab/ac ETEC resistance [32,33].

**Table 1:** Main characteristics of the genes involved in the host’s resistance to ETECs.

Gene	Gene Name	Study Type	Function	Location	Pathogen	Reference
*MUC4*	Mucin 4	In Vivo	Coat the epithelial cells on the apical surfaces, providing one of the first lines of defence against pathogens	Chromosome 13	ETEC F4 ab/ac	[34,35]
*MUC13*	Mucin 13	In Vivo		Chromosome 13	ETEC F4 ab/ac	[36]
*MUC 20*	Mucin 20	In Vivo		Chromosome 13	ETEC F4 ab/ac	[37]
*TFRC*	Transferrin receptor gene	In Vivo	Involved in transporting iron from the transferrin protein into the cell	Chromosome 13	ETEC F4 ab/ac	[38]
*CHCF1*	-	In Vivo	Genetic marker	Chromosome 13	ETEC F4 ab/ac	[39]
*ITGB5*	Integrin subunit beta-5	In Vivo	Plays a role in bacterial adhesion	Chromosome 13	ETEC F4 ab/ac	[40]
*FUT1*	Alpha (1)—fucosyltransferase	In Vivo	Glycosphingolipid biosynthesis	Chromosome 6	ETEC F18	[41,42]
*FUT2*	Alpha (2)—fucosyltransferase	In Vivo		Chromosome 6	ETEC F18	[43]

Organising the information gathered so far chronologically, the first and most studied polymorphism in relation to F4ac resistance was an intronic single-nucleotide polymorphism (SNP) in the *MUC4* gene. Although this polymorphism is not the causal factor of the variability in susceptibility to infection [44,45], this *MUC4* SNP can be used as a genetic marker to predict F4ab/ac susceptibility [34,35]. A year later, a study published by Zhang and collaborators [36] revealed the association of the *MUC13* haplotypes with resistance to F4ab/ac ETEC, suggesting the involvement of variants of this gene in infection resistance. Subsequent studies, nevertheless, refuted a direct causal link [46], prompting further research and identifying the *HEG1-MUC13* interval as the most likely candidate genomic region for infection resistance [47]. In line with this study, the gene *CHCF1*, a marker close to the *MUC13* gene, was proposed as a more reliable genetic marker for predicting F4ac receptor expression [47]. A recent pilot study [39] suggests that susceptibility towards ETEC F4ac/ab infection might match with the *CHCF1* genotype, turning it into a promising resistance marker. Other hypotheses targeting other genes have been established in parallel research. Back in the early 2010s, other SNPs within the *MUC20* gene were initially associated with susceptibility to F4ab/ac infection [48]. However, its relationship with the F4 receptor was later questioned, as no significant differences in *MUC20* expression were observed between susceptible and resistant pigs [37]. In the same period, the transferrin receptor gene was pointed out as a candidate, suggesting its involvement in the response to damage or infection, rather than enabling initial adhesion [38].

Even though many genes have been studied and, at some point, been linked to susceptibility to ETEC F4ab/ac, probably due to their proximity to the actual gene involved in disease resistance, recent studies have dismissed mucin- or transferrin-encoding genes, while other targets, *ITGB5* and *CHCF1*, have been postulated [39,40,49,50]. In this sense, the study performed by Wang and colleagues [40] found that animals overexpressing *ITGB5* were more susceptible, while ETEC F4ac adhesion to porcine epithelial cells was significantly reduced when the gene was knocked out using CRISPR/Cas9 editing.

Despite the relevant number of studies performed and the efforts addressed to disclose the basis of this genetic resistance, the mechanism involved in resistance to ETEC F4ab/ac has not yet been fully elucidated to date, nor has the interaction between the host and other factors such as the microbiota, nutrition, management, and environment, which may influence the susceptibility outcome [51].

Genetic resistance associations have also been identified for ETEC F18, where two fimbrial variants are described, F18ab and F18ac. F18ab is usually found in ETEC and Shiga toxin-producing *E. coli* (STEC), responsible for oedema disease, while F18ac is linked to ETEC, causing PWD [52].

The α (1,2)-fucosyltransferase (*FUT1* and *FUT2*) genes, located in chromosome 6, have received particular attention in relation to ETEC F18 resistance [53]. These genes participate in the porcine blood group antigen structures and are also expressed in the small intestine of pigs. Of these two, *FUT1* has been widely studied and reported as a potential candidate for F18 resistance. A polymorphism in the *FUT1* gene, which causes a guanine-to-adenine transition resulting in an amino acid substitution at position 103 (Ala → Thr), has been identified as conferring resistance to ETEC F18 [54]. This polymorphism leads to three different genotypes (AA, AG, and GG), with the AA genotype conferring resistance to ETEC F18 [54]. This demonstrates the critical role of the *FUT1* enzyme in ETEC F18 adhesion to the small intestine, suggesting that *FUT1* could be a useful target for genetic selection [53,55]. Additionally, it has also been suggested that the AA genotype boosts the immune system, conferring disease resistance in pigs carrying this genotype [41]. In a recent study, 179 pigs belonging to the three different genotypes aforementioned were challenged with ETEC F18. It was found that the AA genotype not only exhibited higher survival and reduced clinical signs but also improved growth performance [42].

The *FUT2* gene has also been proposed as a candidate gene involved in ETEC F18 susceptibility. It has been observed that the expression of this gene is lower in pigs that are resistant to F18 ETEC infections, which suggests that downregulation of *FUT2* enhances resistance to F18 [43]. Apart from *FUT* genes, other gene candidates include an antisense RNA called *FUT3-AS1*, which overlaps with the *FUT3* gene in antisense orientation, and which appears to regulate susceptibility to ETEC F18 via epigenetic and post-transcriptional mechanisms [56].

Although new genes and mechanisms have been postulated in ETEC F18 resistance, *FUT1* remains the main gene responsible for encoding the intestinal receptor for ETEC F18 in pigs. Variations in this gene are directly associated with resistance or susceptibility to ETEC F18 infection. The *FUT2* gene is also involved in glycosphingolipid biosynthesis, but its specific role in forming the F18 receptor in pigs requires further investigation [42,43,53,54,55].

### 3.2. Genetic Resistance of Pigs to Enteric Viruses

Genetic resistance of pigs to enteric viruses has emerged as a critical determinant of disease outcome, particularly during the neonatal period when animals are highly susceptible to severe diarrhoea and dehydration [57,58,59]. The host’s genetic background influences not only viral entry and replication in the host but also the immune response against the pathogen, epithelial barrier integrity, and tissue repair processes [57,58,59]. Candidate genes (Table 2) such as *APN* (encoding aminopeptidase N), *AQP3* (aquaporin-3), and *TFF1* (trefoil factor 1) have been implicated in modulating the susceptibility of pigs to porcine enteric coronaviruses, while glycan-binding profiles and tight junction proteins are central to resistance against rotaviruses [57,58,59].

Advances in CRISPR/Cas9-mediated genome editing have enabled the functional validation of these host factors, demonstrating the technical feasibility of producing genetically resistant pig lines [58]. Collectively, these studies highlight the polygenic and multifactorial nature of resistance to enteric viruses, which combines receptor-mediated effects with regulatory networks governing mucosal immunity and epithelial homeostasis.

Transmissible gastroenteritis virus (TGEV) is a classic porcine alphacoronavirus that causes severe enteritis in suckling piglets, with mortality approaching 100% in naïve herds. The virus uses Porcine Aminopeptidase N (pAPN), expressed abundantly on small intestinal enterocytes, as its primary cellular receptor [60]. Structural and mutational analyses identified specific residues within the extracellular domain of pAPN, notably within domain VII and regions between amino acids 717 and 813, as critical for the viral spike protein binding [66,67,68]. The functional requirement of pAPN has been confirmed in vivo by CRISPR/Cas9-mediated knockout of the gene, which made pigs resistant to TGEV infection. Meanwhile, transgenic overexpression of the gene facilitated the infection to otherwise non-permissive cell lines [69,70,71]. Nonetheless, residual infections in APN-null pigs suggests that other receptors, such as epidermal growth factor receptor (EGFR), may also serve as cell receptors for the virus [69]. Thus, the generation of pAPN-null pigs represents a proof-of-concept for resistance breeding against TGEV, although further work is required to evaluate the impact on production traits and potential compensatory pathways.

Porcine epidemic diarrhoea virus is responsible for devastating outbreaks with high mortality in neonatal piglets worldwide [6]. Considering its relevance, several studies have dug into potential genetic factors which can confer resistance against the infection. For many years, pAPN was proposed as a functional receptor, but accumulating evidence shows that PEDV infection can occur in the absence of pAPN, both in cell lines and in APN-null pigs [60,69,71,72].

Instead, PEDV susceptibility appears to be modulated by alternative host factors and regulatory pathways. One of these factors is AQP3, a membrane channel involved in water and glycerol transport and in maintaining epithelial barrier integrity [73]. Functional assays demonstrated that knockdown of *AQP3* significantly increased viral replication, whereas overexpression of the channel reduced viral load [61]. Moreover, a 16 bp insertion in the *AQP3* promoter enhanced transcriptional activity by creating a binding site for the C/EBPα transcription factor, resulting in increased host resistance. In addition, *AQP3* also modulates cytokine expression (IL-6, IL-8, IL-18, IFN-α, IFN-β) and stabilises tight junction proteins such as ZO-1, thereby enhancing the intestinal barrier function during PEDV infection [62].

Another layer of regulation involves epigenetic control of *TFF1*, which codifies a mucosal protective peptide that promotes epithelial restitution. PEDV infection induces hypermethylation of the *TFF1* promoter, leading to reduced expression and enhanced viral replication. Conversely, overexpression of *TFF1* decreases PEDV titres, reduces apoptosis, and supports epithelial proliferation. The transcription factor C/EBPα plays a central role in regulating *TFF1* expression, linking epigenetic mechanisms with the innate immune response [63].

Overall, genetic resistance to PEDV seems to be polygenic and multifactorial, depending less on a single receptor and more on a network of genes regulating mucosal defence, epithelial integrity, and immune signalling.

Porcine deltacoronavirus (PDCoV) is an emerging enteropathogenic coronavirus with zoonotic potential. Similar to TGEV and PEDV, pAPN has been proposed as a functional receptor, supported by gain-of-function studies in non-permissive cells and reduced infection in pAPN-knockout cell lines [74,75,76]. Interestingly, the tropism of PDCoV for jejunal epithelia, the section with higher APN expression [75], may be another proof-of-concept of this hypothesis. However, APN-null pigs remain susceptible to PDCoV [74,76], suggesting the existence of an alternative receptor, yet unidentified.

Porcine rotaviruses (PRVs), particularly the Rotavirus A and Rotavirus C species, are major causative agents for neonatal diarrhoea in pigs and exhibit host range restrictions which are receptor-dependent. The viral attachment relies on the outer capsid protein VP4, particularly the VP8 domain, which recognises sialic acids and histo-blood group antigens (HBGAs) on the host enterocyte surface. The receptor dependency is strain-specific. Some porcine RVs are strictly sialic acid-dependent, while others engage HBGA as primary or alternative receptors [77,78].

Recent studies using porcine intestinal enteroids have shown that HBGA expression strongly influences RV replication, with distinct HBGA phenotypes conferring resistance or susceptibility [79]. Notably, attenuated RV strains often exhibit reduced dependence on HBGA, suggesting that receptor flexibility contributes to viral adaptation and cross-species transmission [79,80,81].

Beyond glycan recognition, cellular junctional proteins such as the Junctional Adhesion Molecule A (JAM-A) and occludins have been implicated as co-receptors, enabling efficient viral entry once the epithelial barrier is compromised [64,65]. Indeed, the integrity of epithelial tight junctions determines pig susceptibility. Thus, the experimental disruption of junctional complexes markedly enhances RV infection, while restoration of barrier function confers protection [82]. Given the rapid epithelial turnover in weaned pigs compared to neonates, differences in regeneration capacity may also contribute to age-related resistance [77]. Together, these findings underscore that genetic resistance to RV infection is multifactorial, involving glycan diversity, junctional protein availability, and host epithelial dynamics.

## 4. Host Immune Mechanisms Involved in Resistance Against Enteric Pathogens

Considering the relevance of the immune response in the prevention and clearance of infections, this review details the current knowledge in immune factors associated with enteric pathogen resilience.

There is a close and direct link between genetic background and immune response stimulation. Therefore, genetic variation impacting immune responses can also influence responses to pathogens beyond the direct relationships described in the previous section. As an example, F4 ETEC genetic resistance results in potential targeting receptors as discussed in the previous section. Van den Broeck and colleagues [83] investigated the response to F4 fimbriae, using a villous adhesion assay with receptor-positive (F4R^+^) and receptor-negative (F4R^−^) animals. The F4R^+^ pigs developed detectable serum IgG and IgA responses, as well as a mucosal IgA response, whereas the F4R^−^ pigs showed little or no humoral immune response. The study concluded that the presence of F4 receptors was necessary for an effective immune response following oral immunisation.

### 4.1. Evidence of Overall Immune Resilience Against Enteric Bacteria

**Figure 1:**
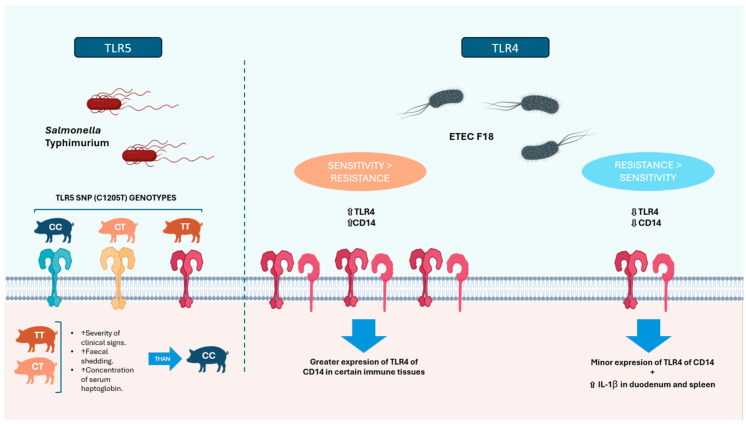
Two different TLR-related mechanisms that could confer host resilience. **Left** [88]: The effect of the *TLR5* SNP, which results in three genotypes with distinct outcomes following *Salmonella* Typhimurium infection. **Right** [89]: The expression levels of *TLR4* and *CD14*, which are associated with differences in sensitivity/resistance in piglets infected with ETEC F18.

The innate response could be crucial in pathogen clearance in early infection. Several studies have compared the differences observed between susceptible and resistant animals in an attempt to identify target genes or early signalling pathways involved in the innate response that could confer resistance to enteric infections. To this end, various methodologies have been put in place to disclose the mechanisms of resilience. For instance, some of these studies have associated immune resistance with specific SNPs in the genome [84,85], while others have used transcriptomic and proteomic approaches to identify pathways and molecules associated with resistance or susceptibility in enteric infections [86,87]. Toll-like receptors (TLRs) are membrane proteins involved in early signalling by identifying pathogen-associated molecular patterns (PAMPs). Polymorphisms in TLRs could have been associated with resistance to *Salmonella* infections [84,85,88]. For instance, SNPs in the *TLR4* gene, involved in the recognition of lipopolysaccharide, have been found to be significantly associated with variability in *Salmonella* shedding. This suggests that these genetic variants could influence the host’s ability to recognise and respond to *Salmonella* infection, thereby affecting bacterial excretion [84]. This idea was also supported by similar results in another study with *TLR4*. Liu et al. [89] reported that higher expression of *TLR4* and *CD14* in certain immune tissues (such as spleen and mesenteric lymph nodes) was associated with susceptibility to ETEC F18 infection, while resistant animals showed increased IL-1β expression in the duodenum and spleen (Figure 1). In contrast, Wu et al. [90] found that the TLR4-mediated signalling pathway was enriched in resistant animals, correlating resistance and *CD14* expression in mononuclear cells. When *CD14* expression was reduced, immune signalling weakened and bacterial adhesion increased. Although it remains unclear whether *TLR4* and *CD14* ultimately strengthen or impair host defence against F18 ETEC, both studies indicate that these molecules play an important role in the immune response, and the discrepancy may reflect differences in pigs’ genetic background, tissues analysed, or immune activation contexts.

Toll-like receptor 5 (TLR5) is a pattern-recognition receptor for bacterial flagellin. Some SNPs that result in amino acid changes, such as TLR5R148L or TLR5P402L, attenuate the response to *Salmonella* Choleraesuis. A study demonstrated differences in the distribution of these two polymorphisms among breeds, which could explain differences in the disease outcome after *Salmonella* challenge [85]. More recently, the effect of an SNP in swine *TLR5* (C1205T) was evaluated in an in vivo challenge in Landrace pigs [88]. It was observed that pigs with the CT and TT genotypes exhibited more severe clinical signs, increased duration in *Salmonella* shedding, and lower serum haptoglobin concentration, an early inflammation marker, suggesting that the CC genotype could be more resilient to the infection, as presented in Figure 1. Furthermore, Dai et al. [91] identified *TLR5* as a candidate gene from a transcriptomic analysis of the duodenum of weaned piglets resistant or susceptible to F18 ETEC. They validated that *TLR5* expression in the duodenum and jejunum was significantly higher in susceptible animals. Functional assays in IPEC-J2 cells showed that *TLR5* overexpression enhanced NF-κB activation and inflammatory responses, leading to increased *E. coli* adhesion, whereas reduced expression limited adhesion. These findings suggest that lower *TLR5* expression may contribute to resistance to ETEC F18.

The early immune signalling could also play a role in resilience to *Salmonella* infection. Thus, variations in IL-1β and IL-8 response have also been observed among animals, indicating that host factors may strongly influence the robustness of this early response [92,93]. *Clostridium perfringens* type C (CpC) is a major pathogen that affects newborn piglets, causing severe to lethal necrotic enteritis. This is characterised by deep, segmental mucosal necrosis and significant haemorrhage of the small intestine [94]. The TLR4/MyD88/NF-κB signalling pathways in the ileum and jejunum have been suggested to be induced by CpC infection, which could provide valuable insights into the innate immune mechanisms involved in regulating piglet diarrhoea caused by CpC [95].

Another approach to disclose potential immune factors involved in disease resilience aims to compare animals with different disease outcomes [96]. A study focused on *Salmonella* infection split animals by their shedding pattern. In this study, piglets were defined as low shedders (LSs) and persistent shedders (PSs) based on *Salmonella* faecal shedding outcome. The analysis of the transcriptome in both shedding phenotypes revealed a higher expression of genes and modules in LSs such as *SLC11A1* (formerly *NRAMP1*), *TLR4*, *CD14* and *CCR1*, some of which have already been mentioned in this review. Other genes that were found to be associated with *Salmonella* for the first time included *SIGLEC5*, *IGSF6,* and *TNFSF13B* [96]. Solute carrier family 11 member 1 (*SLC11A1*) is a host resistance factor that influences susceptibility to intracellular pathogens, such as *Salmonella*. It transports Fe^2+^, Mn^2+^, and Co^2+^ out of the phagosomes, which may deprive vacuolar pathogens of these essential nutrients [97]. Studies in other species have reported that *SLC11A1* is involved in resistance to *Salmonella* infections. Cunrath and Bumann [98] demonstrated that, in mice with different *SLC11A1* alleles, this gene reduced *Salmonella* replication and restricted its access to magnesium, suggesting this to be a key defence mechanism. While no such study has been conducted in pigs, some polymorphisms have been identified in the porcine *SLC11A1* gene [99].

Recently, several studies have been conducted to compare the immune response in susceptible and resistant animals to ETEC. These are typically transcriptomic and proteomic analyses that attempt to analyse the response at a molecular level, investigating whether it differs between resistant and susceptible animals. This includes searching for potential genes and molecules that may be related to the receptor or involved in ETEC resistance. One of such studies examined the response in 40 *Sutai* pigs belonging to two groups (resistant or susceptible according to their *FUT1* genotype, as confirmed by adhesion assays). In this study, the proteins with higher expression in the resistant animals were transferrin (TF), collapsin response mediator protein 2A-like protein, and ribosome protein SA (RPSA). Conversely, susceptible animals exhibited higher expression of vinculin (VCL), beta-actin (ACTB), 27 kDa heat shock protein (HSP27), and cardiac alpha-actin (ACTC1). After all, transferrin was proposed as the most plausible candidate protein related to resistance against F18 ETEC infection, given its physiological function [86].

### 4.2. Immune Mechanisms of Host Resistance to Viral Enteric Infections in Pigs

Viruses are recognised through pattern recognition receptors (PRRs) such as TLRs, RIG-I-like receptors (RLRs), NOD-like receptors (NLRs), and C-type lectin receptors (CLRs), which detect PAMPs and trigger downstream interferon (IFN) and cytokine responses [100,101,102]. The resulting activation of interferon signalling induces a broad antiviral state mediated by interferon-stimulated genes (ISGs) including *OAS1*, *PKR*, *MX1*, *ISG15*, and *ZAP*, which act to inhibit viral replication [103,104].

Among these, type I interferons (IFN-α/β) play a central role by activating the JAK–STAT signalling pathway, leading to phosphorylation of STAT1/STAT2 and formation of the ISGF3 complex with IRF9, which translocates to the nucleus to upregulate ISGs [105,106]. In parallel, the type II interferon (IFN-γ), primarily secreted by NK and T cells, enhances macrophage activation and antiviral responses [107,108]. Adaptive immunity, mediated by virus-specific B and T lymphocytes, reinforces this early innate response, contributing to long-term protection and immunological memory [109,110,111].

Transmissible gastroenteritis virus infection activates PRRs such as retinoic acid-inducible gene I (*RIG-I*), melanoma differentiation-associated gene 5 (*MDA5*), and *TLR3*, and activates TANK-binding kinase 1 (*TBK1*) and interferon regulatory factor 3 (*IRF3*), leading to the induction of type I interferons (IFN-α/β) [106,112,113]. These cytokines initiate the JAK–STAT signalling cascade, forming the ISGF3 complex (STAT1–STAT2–IRF9), which drives the transcription of interferon-stimulated genes (ISGs) [105,114]. Although TGEV partially suppresses early IFN production, infected cells still exhibit phosphorylated STAT1 and increased ISG expression, confirming a functional antiviral response [114].

Among ISGs, IFIT3 plays a key antiviral role. Transmissible gastroenteritis virus infection markedly upregulates IFIT3, which interacts with TBK1 and STAT1 to enhance their phosphorylation and stimulate the transcription of IFN-β, MX1, MX2, OAS1, and ISG15. Overexpression of IFIT3 reduces viral titres, whereas its silencing enhances replication, confirming its antiviral function dependent on the interferon pathway [106].

Another essential mechanism is the activation of the NLRP1 inflammasome. TGEV infection induces caspase-1 cleavage and secretion of IL-1β and IL-18 while disrupting the inhibitory interaction between porcine NLRP1 (pNLRP1) and DPP9. Activated pNLRP1 restricts TGEV replication and upregulates ISGs such as ISG15 and OASL, linking inflammasome activation to antiviral signalling. NLRP1 itself acts as an interferon-stimulated gene, further amplifying antiviral responses [115]. Additionally, TGEV triggers NF-κB activation and regulated cell death processes that contribute to limiting viral dissemination [104].

Similar to what occurs with TGEV, the first barrier to PEDV infection in pigs relies on early detection of viral components by PRRs such as RIG-I, MDA5, and TLR3/7, which activate adaptor proteins including MAVS and TRIF. This triggers phosphorylation of TBK1 and IRF3, along with NF-κB activation, leading to induction of type I (IFN-α/β) and type III (IFN-λ) interferons and proinflammatory cytokines [104,116]. Type I IFNs rapidly induce an antiviral state, while type III IFNs act mainly at mucosal surfaces, sustaining ISG expression in intestinal epithelial cells [117].

Interferons engage the JAK–STAT pathway, forming the ISGF3 complex that drives transcription of ISGs, including MX1/2, OAS, PKR, IFITs, and ISG15, which inhibit viral replication. Inactivation of major PEDV interferon antagonists (NSP1, NSP15, NSP16) restores a robust IFN response, limiting viral replication and attenuating infection in piglets, highlighting the importance of rapid, intact interferon signalling [118].

However, PEDV uses multiple viral proteins to suppress the host interferon response: NSP1 inhibits IFN production by degrading transcriptional cofactors and blocking IRF3 and NF-κB, while NSP3, NSP5, NSP15, and NSP16 disrupt antiviral signalling by removing activating ubiquitin chains, cleaving immune adaptor proteins, degrading viral RNA intermediates, or mimicking host mRNA to evade detection [119,120].

Other host factors also contribute to resistance. IRF8 is upregulated during infection, enhancing IFN-β production and inducing apoptosis in infected epithelial cells, with promoter hypomethylation mediated by AP-2α increasing transcription in resistant pigs [121]. Additionally, PEDV exploits exosomes containing viral RNA and N protein to enable antibody-resistant transmission and viral persistence [122].

Porcine deltacoronavirus (PDCoV) and swine acute diarrhoea syndrome coronavirus (SADS-CoV) both infect intestinal epithelial cells and rely on evasion of host antiviral defences to establish infection. In both viruses, host resistance primarily depends on interferon (IFN)-mediated innate immunity, which is counteracted by the conserved 3C-like protease NSP5. In PDCoV, NSP5 cleaves several host immune regulators: it disrupts NF-κB activation via NEMO, degrades STAT2 to suppress ISG expression, and cleaves the antiviral effector IFIT3, thereby blocking MAVS–TBK1–IRF3 signalling [123]. Similarly, SADS-CoV NSP5 inhibits IRF3 phosphorylation, NF-κB, and STAT1/STAT2 activation, resulting in reduced IFN-β and ISG15 expression [124]. In addition, PDCoV NSP5 targets histone deacetylase 2 (HDAC2), impairing ISG induction and potentially exacerbating inflammation, whereas SADS-CoV NSP5 cleaves DCP1A, disabling its ability to enhance IFN signalling—a mechanism conserved among coronaviruses including PDCoV and SARS-CoV-2 [124,125].

Both viruses also use their nucleocapsid (N) proteins to interfere with RIG-I ubiquitination, further limiting IFN-β production [104,126]. PDCoV additionally employs accessory proteins NS6 and NS7a to inhibit IFN-β transcription by blocking recognition of viral RNA and preventing IRF3 activation, whereas such accessory factors have not been described for SADS-CoV.

As described above for porcine enteric coronaviruses, the porcine intestinal epithelium elicits a rapid antiviral response against rotaviruses through recognition of viral double-stranded RNA by pattern recognition receptors (PRRs) such as TLR3, RIG-I, and MDA5. Upon activation, these sensors trigger the MAVS–TBK1–IRF3 signalling cascade, leading to the production of type I and type III IFNs and the induction of numerous ISGs that restrict viral replication [110].

Type I IFNs (IFN-α/β) act systemically to induce an antiviral state, whereas type III IFNs (IFN-λ) play a predominant role at mucosal surfaces, providing sustained protection of intestinal epithelial cells. The IFN-λ system is particularly relevant in the porcine gut, where high receptor expression in enterocytes enables targeted antiviral defence with minimal inflammation [127]. Age-dependent maturation of the intestinal immune system further influences susceptibility. In neonatal piglets, reduced expression of PRRs and IFN-λ receptors correlates with higher viral replication and disease severity, whereas older pigs exhibit stronger interferon responses and more efficient control of infection [127].

Overall, porcine resilience to enteric viruses depends on rapid interferon signalling, ISG activation, and controlled inflammatory responses in the intestinal epithelium. Yet, these viruses counteract host defences through protease-mediated degradation of antiviral proteins, interferon suppression, and epigenetic modulation of immune regulators. The intestinal epithelium thus serves as a key immune hub where the balance between antiviral defence and viral evasion determines infection outcomes, offering valuable insights for developing improved vaccines and antiviral strategies.

### 4.3. The Role of Non-Coding RNAs in Immune-Mediated Disease Resistance

Non-coding RNAs participate in the regulatory process of gene expression [128,129] and could be involved in infectious disease resistance [130]. Wang et al. [131] studied immune responses in seven-day-old piglets infected with CpC, classifying them as resistant or susceptible based on clinical severity. The study identified 53 microRNAs differentially expressed between resistant and susceptible pigs, with predicted target genes enriched in immune and signalling pathways such as ErbB, MAPK, Jak-STAT, and inverse correlation analysis, suggesting that specific microRNAs such as miR-7134-5p (*NFATC4*), miR-500 (*ELK1*, *HSPA2*, *IL7R*), and miR-92b-3p (*CLCF1*) may regulate key immune genes, potentially serving as biomarkers or functional modulators of host defence. These results indicate that CpC infection altered ileal microRNA expression, influencing genes that determine resistance or susceptibility.

Other studies have focused on long non-coding RNAs (lncRNAs), as they are known to regulate immune and inflammatory responses. Huang et al. [87] compared the expression of lncRNAs between susceptible and resistant pigs and observed that some lncRNAs seemed to regulate key immune genes, such as *TLR8*. They concluded that piglets may become resistant by avoiding excessive inflammatory damage through modulation of cytokine expression, whereas susceptibility may involve an excessive response or aberrant expression. Subsequently, they conducted a genome-wide analysis of changes in DNA methylation and gene expression in CpC-resistant and -susceptible piglets. This analysis identified *LBP*, *TBX21,* and *LCN2* as potential candidate genes involved in the response to CpC infection. Further research is needed to confirm the role of these genes in resistance, but they could be a promising target for breeding strategies [87].

Eventually, transcriptomic studies have also been performed to determine the systemic immune regulation in key organs such as the spleen. Yan et al. [132] identified differentially expressed lncRNAs between susceptible and resistant animals. Two of these were found to be involved in regulating immune and inflammatory pathways. These lncRNAs may modulate the expression of immune genes, playing a role in the systemic immune response to CpC infection and determining whether animals are susceptible or resistant. These results suggest that differences in immune responses may confer resistance to CpC infections, either through increased innate immune molecules or a more cautious response. Further research is needed to identify the main genes, pathways, and molecules involved in these responses, but existing studies provide extensive information about the most promising targets.

In summary, resistance may depend on the correct pattern and regulation of gene expression rather than simply having higher expression levels across the board. Further studies are required to understand these complex mechanisms and also the main differences among pig breeds.

## 5. The Potential Ability of Commensal Beneficial Bacteria to Outcompete Intestinal Pathogens

**Figure 2:**
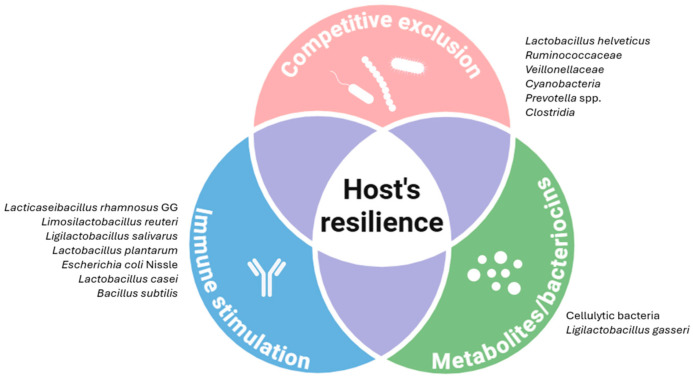
Summary of the main microbiota-mediated mechanisms that contribute to host resilience. The figure illustrates (i) competitive exclusion of pathogens through niche occupation and resource competition, (ii) immune stimulation via modulation of innate and adaptive responses, and (iii) production of metabolites and bacteriocins that enhance barrier protection and suppress pathogen growth. Representative bacterial species associated with each mechanism are shown.

The intestinal microbiota of pigs plays a pivotal role in maintaining gut homeostasis and in shaping host resistance to enteric infections. Commensal bacteria not only contribute to digestion and nutrient absorption but also function as a critical line of defence against pathogens using different strategies which are outlined below. Figure 2 summarises the mechanisms by which this community of bacteria controls the colonisation and overgrowth of harmful bacteria.

### 5.1. Competitive Exclusion

A major role of microbiota in pathogen regulation is summarised under the term competitive exclusion, which refers to the ecological principle that beneficial well-established microbes can prevent harmful or invading microbes from colonising the same environment because they compete for limited resources and physical space [133]. It is well-known that the microbiota and host shape the gut environment (oxygen and nutrient availability) to restrict colonisation by gut pathogens [134]. In addition, commensal bacteria compete with bacteria and viruses for epithelial attachment sites, thereby reducing bacteria or viral entry [135,136,137]. Some bacteria, such as *Lactobacillus helveticus*, can express surface layer proteins (SLPs), providing bacterial adhesion to host cells which can inhibit or hinder the binding of some pathogens. Under in vitro assays with cell lines, the SLPs from *L. helveticus* were able to inhibit the binding of ETEC and *Salmonella* Typhimurium [138]. *E. coli* Nissle (EcN), for example, directly binds human rotavirus particles and prevents their attachment to porcine epithelial cells [139].

In recent years, metagenomics has enabled us to understand much better the role of the microbiota in competitive exclusion mechanisms against enteric pathogens. Different studies have revealed the influence of the microbiota composition in *Salmonella* colonisation resistance or *Salmonella* shedding by infected pigs [140,141,142]. *Clostridia* members and cellulolytic bacteria, such as *Ruminococcaceae*, seem to play a potential role in colonisation resistance to *Salmonella* as they have been found to be more abundant in low/non-shedders and healthy animals [142]. Other bacteria such as *Prevotella* genus, *Veillonellaceae,* or *Cyanobacteria* have also been proposed to play a positive role [141,142]. On the contrary, *Enterobacteriaceae* members and some microaerophilic and aero-tolerant taxa, such as *Lactobacillaceae* or *Pasteurellaceae,* are usually found in the gut when inflammatory responses take place [140,141,142]. Altogether, these results suggest that microbiomes enriched with anaerobes may alleviate or prevent *Salmonella* gut colonisation and faecal shedding. Conversely, a less mature and diverse microbiome can interfere with the host’s resilience to the infection.

The infection by *Clostridioides difficile* (formerly *Clostridium difficile*) can cause fatal gastroenteritis. However, the period during which colonisation can occur is relatively short, as colonisation by *C. difficile* declines as the animal’s age increases [143]. Proctor and collaborators [144] observed that the incidence of clinical signs associated with *C. difficile* infection declined rapidly after four days of age and was no longer observed after ten days, indicating a clear transition period during the first week of life, in which piglets shift from being susceptible to largely resistant. Microbial diversity could be behind resistance to *C. difficile* colonisation [144,145], but there is insufficient evidence to identify the key microbial taxa or communities responsible for this resistance or to determine the extent to which other factors, such as environmental exposure, antibiotic use, diet, and rearing conditions, may contribute [143,144,145].

### 5.2. Metabolites and Bacteriocins to Inhibit Pathogen Colonisation

The mechanisms of competitive exclusion go beyond the mere presence of beneficial bacteria or competition for attachment sites. The products of their metabolism and other compounds (i.e., bacteriocins) produced by these microorganisms also contribute to this phenomenon. Cellulolytic bacteria are major short-chain fatty acid (SCFA) producers. For instance, acetate protects epithelial cells from PEDV-induced damage by upregulating tight junction proteins such as ZO-1 and activating the PI3K/Akt pathway [146]. Besides SCFA, there are a plethora of antimicrobial compounds produced by bacteria and archaea [147,148]. As an example, the bacteriocin gassericin A, produced by *Ligilactobacillus gasseri*, exhibits anti-diarrhoeal and antiviral properties through the modulation of epithelial signalling. This circular peptide interacts with enterocyte membranes, promoting fluid absorption and reducing secretion, thus conferring resistance to diarrhoea in early-weaned piglets [149].

### 5.3. The Stimulation of the Immune System by the Commensal Microbiota

Last but not least, there is a strong positive interaction between the gut commensals, particularly beneficial bacteria with probiotic properties, and the enhancement in the immune response [150]. The administration of the *Lactobacillus casei* probiotic strain favoured mucosal integrity, tight junction protein expression, and immune factor production during ETEC F4 infection [151]. Beyond lactic acid bacteria, Luise et al. [152] demonstrated that dietary supplementation with *Bacillus subtilis* improved the intestinal immune response and mitigated infection severity in weaned pigs challenged with ETEC F4ac.

Several specific probiotic strains have demonstrated stronger efficacy against viral infections in pigs, particularly coronaviruses. Studies with *Ligilactobacillus salivarius* and *Limosilactobacillus reuteri* stimulated the expression of antiviral cytokines while downregulating pro-inflammatory mediators during PEDV infection [153,154]. Don and colleagues [136] revealed that the treatment with *L. salivarius* and its impact on PEDV alleviated endoplasmic reticulum stress and promoted cellular resistance via activation of the FAK/PI3K/Akt signalling axis. Similarly, *Leuconostoc mesenteroides* strains isolated from kefir grains exhibited prophylactic, therapeutic, and direct inhibitory effects against PEDV in vitro, partly by upregulating interferon-stimulated genes such as MX1, ISG15, and OAS1 [155].

Although less extensively studied than coronaviruses, there is also evidence for microbiota and rotaviruses. *Lacticaseibacillus rhamnosus* GG (LGG) and *Lactobacillus plantarum* strains have been shown to decrease viral attachment and replication while enhancing mucosal immune responses [135,156]. EcN 1917 may support rotavirus neutralisation in the intestinal lumen by B-cell activation and IgA production [139]. Studies in gnotobiotic and neonatal pig models further demonstrated that lactic acid bacteria supplementation increases survival, reduces viral shedding, and ameliorates histopathological lesions [156].

Moreover, prebiotics can act synergistically with probiotics to enhance antiviral efficacy. For instance, rice bran supplementation improved the colonisation and activity of LGG and EcN in gnotobiotic pigs, strengthening epithelial integrity, increasing IgA levels, and providing complete protection against rotavirus-induced diarrhoea [110]. Despite these promising outcomes, the direct influence of gut microbiota composition on porcine rotavirus infections remains poorly characterised, underscoring the need for further research into microbiome–virus interactions in pigs.

## 6. Development of Disease Resilience in Pigs: Current Strategies and Future Prospects

### 6.1. Near-Future Approaches in Genome Editing, Immune Resilience, and Microbiome Profiling

Over the past decade, advances in genome editing technologies have opened new avenues for enhancing disease resilience in pigs. The targeted modification of specific genes has made it possible to develop animals with increased resilience to particular pathogens, thus providing a proof of concept for genetic approaches to disease control. For instance, the CD163 gene, which encodes a viral fusion receptor in macrophages, was successfully disrupted in pig zygotes using CRISPR/Cas9, resulting in animals resistant to PRRSv [17]. Moreover, similar gene editing strategies have produced transgenic pigs protected from classical swine fever virus infection, with reduced replication, milder clinical signs, and heritable resistance [18]. These studies highlight the potential of both conventional breeding and genome editing strategies to reduce susceptibility to infectious diseases, offering promising tools for understanding factors impacting pig health at the genetic level. However, relying on gene editing for providing resistance to specific pathogens has drawbacks, as this approach could inadvertently compromise broader immune competence. For example, disrupting a receptor to block one virus could reduce recognition pathways important for defending against unrelated pathogens. As an alternative, selective breeding based on naturally resilient genotypes could promote resistance traits while maintaining overall herd robustness. Ideally, these approaches rely on epidemiogenetics modelling strategies, able to provide a broader perspective by examining how genetic variation across pig populations influences both susceptibility and transmission dynamics of infectious diseases [21]. Indeed, integrating host genetic variation into epidemiological models allows us to identify approaches that not only confer resistance at the individual level but also reduce pathogen spread within herds, thereby offering a sustainable framework for improving overall population health.

Host genetics also plays a pivotal role in shaping immune responses, influencing both vaccine efficacy and post-infection outcomes. Several studies have explored the interplay between host genetics and immune responsiveness to respiratory pathogens such as *Mycoplasma hyopneumoniae* and influenza A virus [157,158,159]. Post-vaccination gene expression analyses have identified genes such as *SPP1*, *ENO3*, and *MYL1* as potential predictors of vaccine response, while genomic regions encompassing *DAB2IP*, *ASAP1*, *CYRIB*, and *GSDMC* were associated with antibody production following *M. hyopneumoniae* vaccination. These findings indicate that combining genetic information with immunological profiling could improve the prediction and optimisation of vaccine responses in pigs.

Recent evidence emphasises the critical role of the gut microbiota in modulating host immunity and overall resistance to enteric infections. Interestingly, host genetics not only influences immune responses but also shapes the gut microbial ecosystem. Indeed, it was recently demonstrated that selective breeding for contrasting enterotypes (*Prevotella*–*Mitsuokella* vs. *Ruminococcus*–*Treponema*) over three generations led to heritable changes in microbial composition and function [160], highlighting the genetic contribution to microbiota structure. Furthermore, next-generation probiotics, such as genetically engineered lactic acid bacteria designed to deliver immunomodulatory molecules or antiviral agents, represent a promising strategy to enhance gut health and disease resilience in pigs [161,162]. Eventually, faecal microbiota transplantation (FMT) has emerged as a powerful tool to restore microbial homeostasis and protect against enteric diseases, with recent human studies exploring the use of sterile faecal filtrates or donor supernatants to confer beneficial effects without transferring live bacteria [163,164]. Collectively, these approaches underscore the combined potential of host genetics, engineered probiotics, and innovative FMT strategies to modulate the microbiome and improve resistance against enteric infections.

Further research is needed to identify new genes or markers involved in disease pathogenesis. Nevertheless, some of the genes or markers mentioned throughout this article could be used as candidates in breeding programmes to confer disease resilience, either by compromising the pathogen’s ability to cause disease or by enhancing the host’s immune response. In addition, growing evidence highlights the role of the gut microbiota in modulating immune function and vaccine responsiveness. Therefore, integrating host genetic, immunological, and microbiota data could provide a more comprehensive approach to improving disease resilience in pigs.

### 6.2. Epigenomics Support in Disease Resilience

Mathematical models integrating host genetics and disease transmission dynamics offer powerful tools for predicting the population-level impact of genetic selection for disease resistance. For parasitic infections such as *Ascaris suum*, epidemiogenetic studies have revealed complex patterns of cross-transmission between humans and pigs, with focal transmission and effective population size influencing parasite genetic diversity [165]. These models can inform breeding strategies by predicting how changes in allele frequencies will affect disease prevalence and persistence within and between herds. For PRRSv, the WUR0000125 SNP has been extensively studied for its effects on viral load and clinical outcomes. However, recent transmission studies revealed that this resilience SNP had no apparent effect on pigs’ infectivity and susceptibility in controlled transmission trials [166], highlighting the complex relationship between individual genetic resistance and population-level transmission dynamics.

Recent methodological advances have opened new possibilities for estimating genetic effects underlying disease transmission dynamics. While traditional quantitative genetic approaches focus exclusively on individual disease susceptibility or resistance, novel Bayesian inference methods now enable simultaneous estimation of genetic parameters for three distinct epidemiological host traits: susceptibility (propensity to become infected), infectivity (propensity to transmit infection to others), and recoverability (propensity to recover or die) [167]. These methods can accommodate the complex dynamic interdependence between observable disease phenotypes and underlying unobservable epidemiological traits, using individual disease records typically available from field studies or challenge experiments. Furthermore, genetic selection targeting infectivity, in addition to susceptibility, may reduce the risk of inadvertently selecting for tolerant super-spreaders—individuals that do not show clinical signs but efficiently transmit pathogens to others.

An important consideration when implementing genetic selection for disease resilience is the potential for pathogen evolutionary responses to host genetic changes. While genetic selection for host resistance offers promising avenues for disease control, pathogens have demonstrated remarkable capacity to adapt to interventions, as evidenced by widespread antibiotic resistance. However, recent epidemiogenetic modelling suggests that disease eradication through genetic selection remains achievable if selective pressure on host resistance is sufficiently strong and sustained [168]. The key principle is that pathogen evolution requires ongoing transmission; interventions that rapidly reduce transmission below the epidemic threshold (R_0_ < 1) can achieve eradication before resistant pathogen strains emerge. This argues for implementing strong, multi-generational selection for polygenic resistance rather than gradual improvement, combined with other management interventions to maximise the rate of prevalence reduction. Selection for broad-spectrum, polygenic resistance targeting multiple host defence mechanisms simultaneously should minimise the probability of pathogen adaptation, as the pathogen would need to overcome multiple independent barriers. Nevertheless, surveillance for pathogen evolutionary changes should be integrated into breeding programmes, particularly when selecting for major-effect resistance genes or receptors, to allow timely adjustments in selection strategies if pathogen adaptation is detected.

## Figures and Tables

**Table 2:** Main characteristics of the genes involved in the host’s resistance to viruses.

Gene	Gene Name	Study Type	Function	Location	Pathogen	Reference
*APN*	Amino peptidase N	In Vivo	Primary cellular receptor for TGEV	Chromosome 7	TGEV	[60]
*AQP3*	Acuaporin-3	In Vivo	Water and glycerol transport and maintenance of epithelial barrier integrity	Chromosome 10	PEDV	[61,62]
*TFF1*	Trefoil Factor 1	In Vivo	Required for the growth of porcine intestinal cells	Chromosome 13	PEDV	[63]
*JAM-A*	Junctional Adhesion Molecule A	In Vitro	Tight-junction protein that regulates epithelial barrier integrity	Chromosome 4	PRV	[64,65]

## Data Availability

No new data were generated for this review.

## Supplementary Materials

The following supporting information can be downloaded at https://www.mdpi.com/article/10.3390/genes17010067/s1, Figure S1: Literature search strategy for studies on swine host resilience and enteric disease. The search combined controlled vocabulary and free-text terms across three concept blocks: genetics, immune mechanisms, and microbiome-mediated resistance. All search codes were kept intact, and the complete search strings are provided in this figure.

## Abbreviations

The following abbreviations are used in this manuscript:

CpC	*Clostridium perfringens* type C
ETEC	Enterotoxigenic *Escherichia coli*
IL	Interleukin
NSP	Nonstructural Protein
PDCoV	Porcine Deltacoronavirus
PEDV	Porcine Epidemic Diarrhoea Virus
PRRSv	Porcine Reproductive and Respiratory Syndrome Virus
RV	Rotavirus
SADSCoV	Swine Acute Diarrhoea Syndrome Coronavirus
SNP	Single-Nucleotide Polymorphism
TGEV	Transmissible Gastroenteritis Virus
TLR	Toll-Like Receptor
VP	Viral Protein
