# Supplementary material for: Factors Involved in Host Resilience to Enteric Infections in Pigs: Current Knowledge in Genetic, Immune, and Microbiota Determinants of Infection Resistance
Source: Genes (Basel). 2026 Jan 6;17(1):67. doi: 10.3390/genes17010067 (PMC12840623; doi:10.3390/genes17010067)
Supplement: Supplementary file 1 [file genes-17-00067-s001.zip › genes-4043025-supplementary.pdf]

1. Genetics

```
(("Swine"[Mesh] OR pigs*[tiab] OR porcine[tiab])
 AND (resilien*[tiab] OR toleran*[tiab] OR robust*[tiab] OR "genetic resistance"[tiab] OR resist*[tiab])
 AND (enteric[tiab] OR intestinal[tiab] OR gut[tiab] OR gastrointestinal[tiab] OR diarrh*[tiab])
 AND (genetic*[tiab] OR genomic*[tiab] OR heritab*[tiab] OR GWAS[tiab] OR QTL[tiab] OR SNP*[tiab] OR polymorph*[tiab])
 AND ("Escherichia coli"[tiab] OR ETEC[tiab] OR Salmonella[tiab] OR "Lawsonia intracellularis"[tiab] OR "Brachyspira hyodysenteriae"[tiab] OR "Brachyspira pilosicoli"[tiab] OR "Clostridium perfringens"[tiab] OR rotavirus[tiab] OR PEDV[tiab] OR TGEV[tiab] OR PDCoV[tiab] OR SADS-CoV[tiab] OR coccid*[tiab] OR "Cystoisospora suis"[tiab] OR Eimeria[tiab]))
```

→ 209 results (2000-2025); 39 selected.

2. Immune mechanisms

```
(("Swine"[Mesh] OR pigs*[tiab] OR porcine[tiab])AND (resilien*[tiab] OR toleran*[tiab] OR robust*[tiab] OR resist*[tiab])AND (enteric[tiab] OR intestinal[tiab] OR gut[tiab] OR gastrointestinal[tiab] OR diarrh*[tiab])AND (immune*[tiab] OR "mucosal immun*"[tiab] OR "innate immun*"[tiab] OR "adaptive immun*"[tiab] OR IgA[tiab] OR cytokine*[tiab] OR "disease tolerance"[tiab])AND ("Escherichia coli"[tiab] OR ETEC[tiab] OR Salmonella[tiab] OR "Lawsonia intracellularis"[tiab] OR "Brachyspira hyodysenteriae"[tiab] OR "Clostridium perfringens"[tiab] OR rotavirus[tiab] OR PEDV[tiab] OR TGEV[tiab] OR PDCoV[tiab] OR SADS-CoV[tiab] OR coccid*[tiab] OR "Cystoisospora suis"[tiab] OR Eimeria[tiab]))
```

→246 results (2000-2025); 39 selected.

3. Microbiome-mediated resistance

```
(("Swine"[Mesh] OR pigs*[tiab] OR porcine[tiab])AND (resilien*[tiab] OR toleran*[tiab] OR robust*[tiab] OR resist*[tiab] OR "colonization resistance"[tiab] OR "competitive exclusion"[tiab])AND (enteric[tiab] OR intestinal[tiab] OR gut[tiab] OR gastrointestinal[tiab] OR diarrh*[tiab])AND (microbiota[tiab] OR microbiome[tiab] OR probiot*[tiab] OR prebiot*[tiab] OR synbiot*[tiab] OR "fecal microbiota transplant*"[tiab] OR FMT[tiab])AND ("Escherichia coli"[tiab] OR ETEC[tiab] OR Salmonella[tiab] OR "Lawsonia intracellularis"[tiab] OR "Brachyspira hyodysenteriae"[tiab] OR "Clostridium perfringens"[tiab] OR rotavirus[tiab] OR PEDV[tiab] OR TGEV[tiab] OR PDCoV[tiab] OR SADS-CoV[tiab] OR coccid*[tiab] OR "Cystoisospora suis"[tiab] OR Eimeria[tiab]))
```

→214 results (2000-2025); 25 selected.

Figure S1: Literature search strategy for studies on swine host resilience and enteric disease.

The search combined controlled vocabulary and free-text terms across three concept blocks: genetics, immune mechanisms, and microbiome-mediated resistance. All search codes were kept intact, and the complete search strings are provided in this figure.
